# Physical Properties and Cellular Metabolic Characteristics of 3D Spheroids Are Possible Definitive Indices for the Biological Nature of Cancer-Associated Fibroblasts

**DOI:** 10.3390/cells12172160

**Published:** 2023-08-28

**Authors:** Nami Nishikiori, Kohichi Takada, Tatsuya Sato, Sho Miyamoto, Megumi Watanabe, Yui Hirakawa, Shohei Sekiguchi, Masato Furuhashi, Akira Yorozu, Kenichi Takano, Akihiro Miyazaki, Hiromu Suzuki, Hiroshi Ohguro

**Affiliations:** 1 Department of Ophthalmology, Sapporo Medical University, S1W17, Chuo-ku, Spporo 060-8556, Japan; nami076@yahoo.co.jp (N.N.); watanabe@sapmed.ac.jp (M.W.); 2 Department of Medical Oncology, Sapporo Medical University, S1W17, Chuo-ku, Spporo 060-8556, Japan; ktakada@sapmed.ac.jp; 3 Department of Cardiovascular, Renal and Metabolic Medicine, Sapporo Medical University, S1W17, Chuo-ku, Spporo 060-8556, Japan; sato.tatsuya@sapmed.ac.jp (T.S.); furuhasi@sapmed.ac.jp (M.F.); 4 Department of Cellular Physiology and Signal Transduction, Sapporo Medical University, S1W17, Chuo-ku, Spporo 060-8556, Japan; 5 Department of Oral Surgery, Sapporo Medical University, S1W17, Chuo-ku, Spporo 060-8556, Japan; miyamoto.sho@sapmed.ac.jp (S.M.); 1989shohei1028@gmail.com (S.S.); amiyazak@sapmed.ac.jp (A.M.); 6 Department of Molecular Biology, Sapporo Medical University, S1W17, Chuo-ku, Spporo 060-8556, Japan; ayorozu@sapmed.ac.jp (A.Y.); hsuzuki@sapmed.ac.jp (H.S.); 7 Department of Otolaryngology, Sapporo Medical University, S1W17, Chuo-ku, Spporo 060-8556, Japan; kent@sapmed.ac.jp

**Keywords:** cancer-associated fibroblast, 3D spheroid culture, oral squamous carcinoma, Seahorse bioanalyzer

## Abstract

The current study’s objective was to elucidate some currently unknown biological indicators to evaluate the biological nature of cancer-associated fibroblasts (CAFs). For this purpose, four different CAFs, CAFS1, CAFS2, SCC17F and MO-1000, were established using surgical specimens from oral squamous cell carcinomas (OSCC) with different clinical malignant stages (CAFS1 and CAFS2, T2N0M0, stage II; SCC17F and MO-1000, T4aN2bM0, stage IVA). Fibroblasts unrelated to cancer (non-CAFs) were also prepared and used as controls. Initially, confirmation that these four fibroblasts were indeed CAFs was obtained by their mRNA expression using positive and negative markers for the CAF or fibroblasts. To elucidate possible unknown biological indicators, these fibroblasts were subjected to a cellular metabolic analysis by a Seahorse bioanalyzer, in conjugation with 3D spheroid cultures of the cells and co-cultures with a pancreas ductal carcinoma cell line, MIA PaCa-2. The mitochondrial and glycolytic functions of human orbital fibroblasts (HOF) were nearly identical to those of Graves’-disease-related HOF (GOF). In contrast, the characteristics of the metabolic functions of these four CAFs were different from those of human conjunctival fibroblasts (HconF), a representative non-CAF. It is particularly noteworthy that CAFS1 and CAFS2 showed markedly reduced ratios for the rate of oxygen consumption to the extracellular acidification rate, suggesting that glycolysis was enhanced compared to mitochondrial respiration. Similarly, the physical aspects, their appearance and stiffness, of their 3D spheroids and fibroblasts that were induced effects based on the cellular metabolic functions of MIA PaCa-2 were also different between CAFs and non-CAFs, and their levels for CAFS1 or SCC17F were similar to those for CAFS2 or MO-1000 cells, respectively. The findings reported herein indicate that cellular metabolic functions and the physical characteristics of these types of 3D spheroids may be valuable and useful indicators for estimating potential biological diversity among various CAFs.

## 1. Introduction

Fibroblasts are known to be the cells that are responsible for producing various extracellular matrix (ECM) proteins such as collagen, elastin and others that are integrated into the cell architectures of a wide variety of mesenchymal-related tissues [1,2,3,4,5]. It has been shown that fibroblasts are not only functionally involved in the structural maintenance of tissues but also in the physiological and pathological wound-healing processes of most organs [6,7,8,9]. It has been shown that the biological behaviors of fibroblasts are exclusive and diverse among those originating from different body sites, and these characteristics may be related to adapting to diverse pathophysiological environments that differ depending on the region of the body [5]. Within the field of cancer biology, the functional significance of the tumor surrounding microenvironment (TSM) is that specific fibroblasts that are referred to as “cancer-associated fibroblasts (CAFs)” are known to be the major participant, and this has attracted great interest because of their crucial roles in tumorigenesis, tumor progression and metastasis [10,11,12,13]. For example, it was reported that CAFs enhance in vivo cancer progression after they are co-injected with malignant tumor cells or are administered to the cancerous site [14,15]. However, although the contributions of CAFs to the pathogenesis of cancer are extremely important, as stated above, precise methods for the definition and characterization of CAFs are not available, except for certain markers such as α-smooth muscle actin (αSMA), fibroblast-activating protein-α (FAP) and others [16], presumably due to substantial diversities of the phenotypic and functional aspects of CAFs. In fact, recent transcriptome analyses have revealed that CAFs from different origins have unique gene expression patterns [11,17,18,19,20]. Nevertheless, since such sophisticated methods are not currently easily conducted, simpler methods for the identification and characterization of CAFs are highly desirable in order to achieve a better understanding of the pathophysiological roles of CAFs.

Recent studies revealed that conventional in vitro disease models using two-dimensional (2D) planar cell cultures indicate that their biological characteristics are quite different from those of the in vivo physiological states as compared with in vitro 3D culture models, based on several differences in the profiles of several gene expression, intercellular binding properties, interactions with ECM proteins in both cancerous and non-cancerous tissues [21,22]. It is especially noteworthy that, in the case of solid malignant tumors such as oral squamous cell carcinoma (OCSS), since some gradients of several nutrients, oxygen and other biological molecules must be present within these solid tumor masses, 3D culture methods would be more desirable in terms of replicating such solid tumor mass environments. Because of this, in vitro 3D models may be more suitable for achieving more accurate screening, estimating proper dosages and estimating the efficacies of new candidate antitumor agents [23,24,25,26,27].

Among the various in vitro 3D cell culture methods, a simpler model, a 3D spheroid culture model, has been the most widely used [28,29]. In our recent investigations, to characterize the microenvironments of various tissues, we independently developed unique 3D drop cell culture methods using several non-cancerous cells including human conjunctival fibroblasts (HconF) [30], human orbital fibroblasts (HOF) [31], human scleral stromal fibroblasts (HSclF) [32] and human scleral fibroblasts (HSSF) [33], and Graves’-disease-related HOF (GOF) [34], as well as cancerous cells including an A549 lung adenocarcinoma cell line [35], various malignant melanoma (MM) cell lines [36] and various OSCC. As expected, our findings indicated that the biological natures of the 3D spheroid models were markedly different and distinct from those of 2D cultured models even though both were prepared under exactly identical experimental conditions except that the culture plates that were used were different [37]. In addition to such functional diversity between both models, we also found that the 3D spheroid architectures were quite different between non-cancerous and cancerous cells; that is, the former formed a globe-shaped and the latter, a non-globe-shaped 3D spheroid [30,31,38]. We also found that the non-globe-shaped configurations of 3D cancerous spheroids and the cellular metabolic functions, as measured by a Seahorse bioanalyzer, of the originated malignant tumor cells could also be modulated by their malignancy grades, sensitivity to antitumor drugs and other pathological factors [36]. Therefore, based upon these collective observations, we hypothesized that a 3D spheroid configuration and the cellular metabolic state of the originated malignant tumor cells may become useful indicators for the clinical and pathophysiological aspects of malignant tumors, including MM, OSCC and others. Since CAFs are greatly influenced by neighboring malignant tumors and, in fact, it has been suggested that normal fibroblasts could be converted into CAFs within the tumor microenvironment via various mechanisms [39], we rationally speculated that the use of 3D spheroid cultures in conjunction with cellular metabolic measurements might provide additional insights into strategies for the identification and characterizations of various CAFs that originate from different malignant tumors.

Therefore, in the current study, to elucidate the biological significance of 3D spheroid cultures and the cellular metabolic functions of CAFs, we used CAFs obtained from four OSCC patients with clinically different malignant stages. We then examined the physical properties of the 3D CAF spheroids and cellular metabolic functions of the originated CAFs and compared these characteristics among the spheroids derived from four CAFs and spheroids derived from five different normal fibroblasts.

## 2. Materials and Methods

The current study, which was conducted at the Sapporo Medical University Hospital, Japan, was approved by the institutional review board (IRB, registration number 342-3416) according to the tenets of the Declaration of Helsinki and national laws for the protection of personal data. Written informed consent for use of the surgical specimens in the current investigation was obtained from all subjects.

### 2.1. Establishing Cancer-Associated Fibroblasts (CAFs) Related to Oral Squamous Carcinoma (OSCC) and Their 2D and 3D Cell Cultures

CAFs containing tissues adjacent to malignant tumors were separated from surgically dissected specimens from 4 patients with OSCC. The isolated peritumoral tissues containing the CAFs were further minced into 1–2 mm pieces, placed on 100 mm culture dishes and completely submersed in a culture medium composed of DMEM supplemented with 10% FBS, 1% *L*-Glutamine and 1% Antibiotic-Antimycotic. This primary tissue culturing was carried out in a humid incubator at 37 °C with 5% CO_2_ with medium change at 2-day intervals. During these tissue cultures, fibroblasts that proliferated in the surrounding mass of tissues were collected and cultured under the same conditions. When the cell confluency reached 80–90%, the cells were washed with phosphate-buffered saline (PBS), dispersed by a 0.25% EDTA/trypsin treatment and collected by centrifugation at 1500 rpm for 5 min. The cells were resuspended in the same medium and further cultured for use in the current investigation as CAFs. The prepared CAFs originated from four patients with OSCC, each of which were designated as CAFS1, CAFS2, SCC17F and MO-1000, respectively. Based upon the clinical stages, the pathological malignancy grades and the Yamamoto–Kohama (YK) classification of invasion [40] of the originated OSCC tumors were in CAFS1 and CAFS2 (T2N0M0, stage II, moderately differentiated and YK-4C), SCC17F (T4aN2M0, stage IVA, poorly differentiated and YK-4D) and MO-1000 (T4aN2bM0, stage IVA, moderately differentiated and YK-3) (Table 1). Two-dimensional and three-dimensional cell cultures of these four CAFs were prepared across 5 days, essentially using previously described methods using non-cancerous and cancerous cells [31,37,41]. Since the CAFs originated from patients with different ages and genders, their phenotypes may be greatly affected by cellular senescence during their passaging. Therefore, to minimize these possible passaging-induced effects among CAFs, CAFs prepared during 1–2 passagings were used in the current investigation.

As non-CAFs (Table 2), a human orbital fibroblast (HOF), Graves’-disease-related HOF (GOF), human scleral stromal fibroblast (HSSF) and human scleral fibroblast (HSclF) were also subjected to the 3D spheroid culture as above. Alternatively, a human conjunctival fibroblast (HconF) was purchased from ScienCell Research laboratories (Carlsbad, CA, USA).

Briefly, these cells were each cultured in 2D planar culture dishes at 37 °C in HG-DMEM culture medium supplemented with 8 mg/L d-biotin, 4 mg/L calcium pantothenate, 100 U/mL penicillin, 100 μg/mL streptomycin (b.p. HG-DMEM), 10% CS and methylcellulose (Methocel A4M) until reaching approximately 90% confluence. The resulting cells were divided into conventional 2D planar cultures and 3D spheroid drop cultures. The 2D cultured cells were further maintained with medium changes daily until day 5. In the case of generating the 3D spheroids, after washing with a phosphate-buffered saline (PBS), the cells were detached by treatment with 0.25% trypsin/EDTA and resuspended in the culture medium, and approximately 20,000 cells within a 28 μL volume of culture medium were placed into each well of the drop culture plate (#HDP1385, Sigma-Aldrich, St. Louis, MO, USA) (3D/day 0) as described previously [31,41]. Thereafter, half of the culture medium was exchanged with fresh medium in each well daily until day 5 [37,42]. At day 5, both the 2D and 3D cultured cells were collected and used in further analyses as described below.

### 2.2. Co-Cultures of a Pancreatic Ductal Adenocarcinoma Cell Line (MIA PaCa-2) with CAFs or Non-CAFs

A pancreatic ductal adenocarcinoma cell line, MIA PaCa-2 (10^5^ cells, CRL-1420, American Tissue Cell Culture, Manassas, VA, USA), was co-cultured indirectly with 10^5^ cells from each of four different CAFs as prepared above or human conjunctival fibroblasts (HconF) as non-CAFs using a Transwell cell culture insert (ThinCertTM 657641; Greiner Bio-One, Singapore) in DMEM supplemented with 10% FBS for 24 h. The cancerous cells and fibroblasts were then collected after a trypsin EDTA treatment and subjected to a Seahorse cellular metabolic measurement analysis as described below.

### 2.3. Real-Time Analysis of the Cellular Metabolic Functions

The oxygen consumption rate (OCR) and extracellular acidification rate (ECAR) were measured using a Seahorse XFe96 real-time metabolic analyzer (Agilent Technologies, Santa Clara, CA, USA). For the experiments on 2D-cultured fibroblasts alone, 2.0 × 10^4^ cells of HOFs, CAFS1, CAFS2, SCC17F, MO-1000 and HconF were placed in each well of a XFe96 Cell Culture Microplate (Agilent Technologies, #103794-100) the day before the assay and incubated at 37 °C. On the day of the assay, the culture medium was replaced with Seahorse XF DMEM assay medium (pH 7.4, Agilent Technologies, #103575-100) containing 5.5 mM glucose, 1.0 mM sodium pyruvate and 2.0 mM glutamine. The assay plates were then incubated in a CO_2_-free incubator at 37 °C for 1 h prior to the measurements.

For the experiment of co-cultured MIA PaCa-2 with fibroblasts including CAFs or non-CAFs, 1.0 × 10^4^ cells of MIA PaCa-2 were suspended in 50 μL of Seahorse XF DMEM assay medium containing 5.5 mM glucose, 1.0 mM sodium pyruvate and 2.0 mM glutamine and were placed in each well of a Seahorse XF poly-*D*-lysine (PDL)-coated Cell Culture Microplate (Agilent Technologies, #103798-100). The plate was then incubated in a CO2-free incubator at 37 °C for 30 min and centrifuged at 300× *g* for 10 min. After the centrifugation, 130 μL of Seahorse assay medium was added to each well and the plate was further incubated in a CO_2_-free incubator at 37 °C for 1 h prior to the measurement.

The OCR and ECAR were simultaneously measured in an XFe96 extracellular flux analyzer at the baseline and under the following sequential injections of 2.0 μM oligomycin, 5.0 μM carbonyl cyanide-p-trifluoromethoxyphenylhydrazone (FCCP), a mixture of 1.0 μM rotenone and 1.0 μM antimycin A, and 10 mM 2-deoxyglucose (2DG). The values were normalized to the amount of protein, as determined by a BCA protein assay (TaKaRa BCA Protein Assay) in each well after the measurement was completed. Key parameters of mitochondrial and glycolytic functions were calculated as follows: basal OCR = OCR at baseline − OCR after adding R/A; ATP-linked respiration = OCR at baseline − OCR after adding oligomycin; maximal respiration = OCR after adding FCCP − OCR after adding R/A; non-mitochondrial respiration = OCR after adding R/A; basal ECAR = ECAR at baseline − final measurement of ECAR after adding 2DG; glycolytic capacity = ECAR after adding oligomycin − final measurement of ECAR after adding 2DG; non-glycolytic acidification = final measurement of ECAR after adding 2DG.

### 2.4. Morphological Analyses of 3D Spheroids Derived from Four Different CAFs and Non-CAFs

Regarding the morphological aspects of the above-prepared 3D spheroids, their downward and lateral views were observed by phase contrast microscopy as reported in our previous study [31,37,43].

### 2.5. Measurement of the Mechanical Stiffness Properties of 3D Spheroids

To evaluate the mechanical stiffness properties of the 3D spheroids obtained from CAFs and non-CAFs, we used a procedure basically reported in our previous studies [31]. In a typical run, a single living 3D spheroid was compressed to 50% of its diameter over 20 s using a micro spheroid compressor system (MicroSquisher, CellScale, Waterloo, ON, Canada). The force (μN) required and its diameter (μm) when 50% compressed were simultaneously measured by a micro-pressure sensor equipped with a micro monitoring camera. To estimate the mechanical hardness of the 3D spheroid, the index of the required force (μN)/50% diameter (μm) was used.

### 2.6. Trypsin-Induced Dispersion of 3D CAF Spheroids

To compare the trypsin-induced destruction of the 3D spheroids obtained from CAFs as above, the time required for them to be dispersed against a 0.05% trypsin solution was determined using phase contrast microscopy.

### 2.7. Other Methods

Quantitative PCR using specific primers (Table S1) and statistical analyses using the Graph Pad Prism 8 (GraphPad Software, San Diego, CA, USA) were performed as described in a previous report [37]. Scanning electron microscopy (SEM) using a HITACHI S-4300 microscope was operated at 5 keV (the detector features 1280 × 960 pixel) as basically reported in our previous study [37]. For estimation of the statistical difference between study groups, the Student’s *t*-test for two group comparison or one-way ANOVA followed by a Tukey’s multiple comparison test were used.

## 3. Results

**Figure 1:**
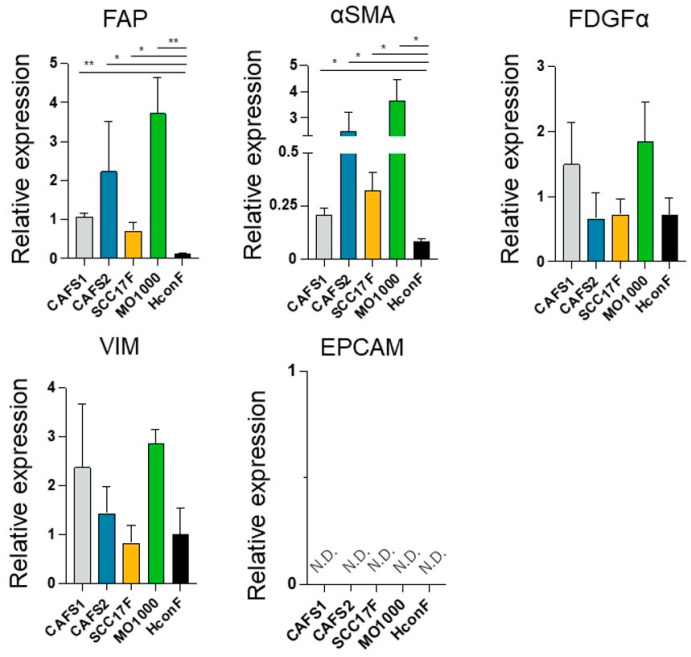
mRNA expression of several positive and negative markers for CAFs and fibroblasts among 4 different CAFs and non-CAFs. We prepared 4 different CAFs including CAFS1, CAFS2, SCC F17 and MO-1000 and a representative non-CAF, human conjunctival fibroblast (HconF). The mRNA expressions of CAF makers (FAP, fibroblast activating protein; α-SMA, a smooth muscle actin), fibroblast markers (PDGFRα, platelet-derived growth factor receptor α; and VIM, vimentin) and their negative marker (EpCAM, epithelial cell adhesion molecule) were evaluated by a qPCR. All experiments were performed in triplicate. * *p* < 0.05, ** *p* < 0.01.

In the current study, to determine whether CAFs from various sources share common biological characteristic or whether there are differences, we prepared four different CAFs termed CAFS1 and CAFS2, which were associated with highly differentiated OSCC, and MO-1000 and SCC F17, which were associated with poorly differentiated OSCC. In addition, since there are fibroblasts that are not related to cancers (non-CAFs), we also used human conjunctival fibroblasts (HconF), human orbital fibroblasts (HOF), Graves’-related-HOF (GOF), human scleral stromal fibroblasts (HSSF) and human scleral fibroblast (HSclF). Among these, the HconF fibroblasts were commercially available, and the others were established in our laboratory. Initially, to verify the identities of these cells, the mRNA expression of CAF makers (FAP, fibroblast activating protein; and α-SMA, a smooth muscle actin), fibroblast markers (PDGFRα, platelet-derived growth factor receptor α; and VIM, vimentin) and the corresponding negative markers (EpCAM, epithelial cell adhesion molecule) were measured. As shown in (Figure 1), as compared with a representative non-CAF, HconF, the mRNA expressions of the CAF markers, *FAP* and α*SMA*, of all four CAFs were significantly upregulated and those levels were higher in CAFS2 and MO-1000. In contrast, the gene expressions of the fibroblast makers, *PDGFR*α and *VIM*, or their negative marker, *EpCAM*, were detected or not detected (N.D.), respectively, and these levels were not statistically significant among the five fibroblasts. Based upon these results, the four established fibroblasts used in this study, CAFS1, CAFS2, SCC17F and MO-1000, were confirmed to be CAFs.

**Figure 2:**
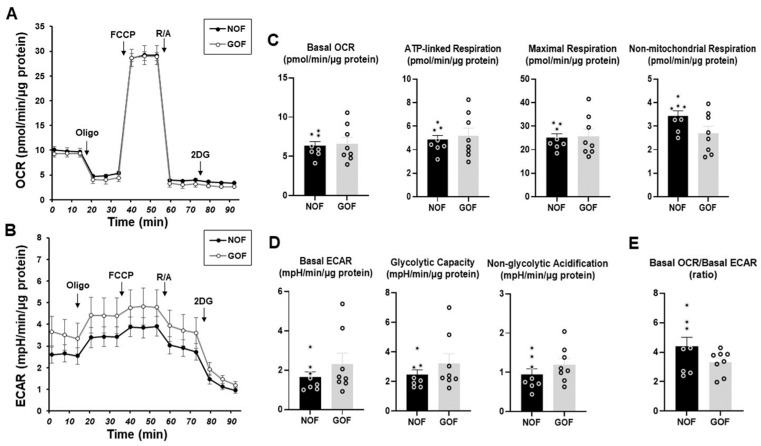
Measurement of the mitochondrial and glycolytic functions of human orbital fibroblasts (HOFs). A Seahorse real-time metabolic function analysis of 2D-cultured normal human orbital fibroblasts (NOF) and Graves’-disease-related HOF (GOF). OCR (panel (**A**)) and ECAR (panel (**B**)) were measured at baseline and those with injections of sequential supplementation with a complex V inhibitor, oligomycin (Oligo), a protonphore, carbonyl cyanide-p-trifluoromethoxyphenylhydrazone (FCCP), complex I/III inhibitors, rotenone/antimycin A (R/A) and a hexokinase inhibitor, 2-deoxyglucose (2DG). Key parameters of mitochondrial functions and glycolytic functions are shown in panels (**C**,**D**), respectively. Basal OCR/basal ECAR ratio is shown in panel (**E**). All experiments were performed using fresh preparations (*n* = 8). Data are expressed as mean or individual values and all error bars are displayed as SEM.

To clarify the unidentified biological characteristics among the CAFs established from different sources of tumor specimens, we compared the cellular metabolic functions among four different CAFs using a Seahorse bioanalyzer. In advance, to determine the mitochondrial and glycolytic functions of non-CAFs, fibroblasts of HOF and Graves’-disease-related HOF (GOF), which are non-CAFs with the same origin but different pathological conditions, were compared. Various indices related to both the OCR and ECAR, mitochondrial and glycolytic functions, respectively, of the HOF and GOF were essentially identical with each other, suggesting that the cellular metabolic states of the non-CAFs from the same origin were quite stable and not significantly influenced by non-cancerous pathologic conditions such as inflammation (Figure 2).

**Figure 3:**
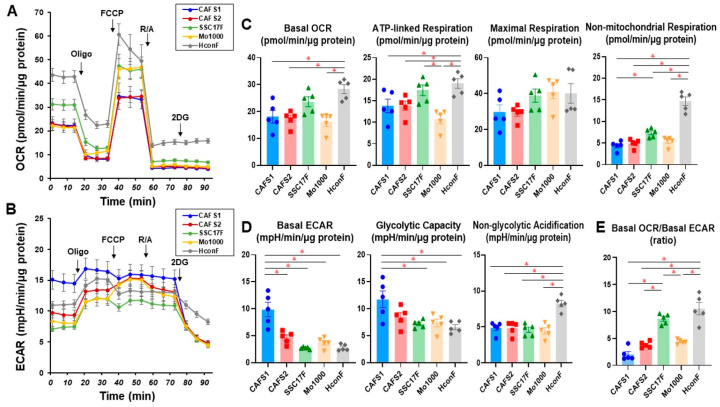
Measurement of the mitochondrial and glycolytic functions of four CAFs and HconF, a non-CAF. A Seahorse real-time metabolic function analysis of 2D-cultured CAFs (CAFS1, CAFS2, SCC17F and MO-1000) and HconF as a representative non-CAF. OCR (panel (**A**)) and ECAR (panel (**B**)) were measured at baseline and those with injections of sequential supplementation with a complex V inhibitor, oligomycin (Oligo), a protonphore, carbonyl cyanide-p-trifluoromethoxyphenylhydrazone (FCCP), complex I/III inhibitors, rotenone/antimycin A (R/A) and a hexokinase inhibitor, 2-deoxyglucose (2DG). Key parameters of mitochondrial functions and glycolytic functions are shown in panels (**C**,**D**), respectively. Basal OCR/basal ECAR ratio is shown in panel (**E**). All experiments were performed using fresh preparations (*n =* 6). Data are expressed as mean or individual values and all error bars are displayed as SEM. * *p* < 0.05.

In contrast, the levels of the basal OCR and ATP-linked respiration of the CAFs were substantially or relatively lower than those of HconF, and among the CAFs, the basal ECAR and glycolytic capacity were both markedly increased in CAFS1 as compared with the others and HconF (Figure 3). In addition, non-mitochondrial respiration and non-glycolytic acidification in these four CAFs were significantly reduced, and the basal OCR/ECAR ratios in CAFS1 and CAFS2 were significantly decreased as compared with a representative non-CAF, HconF (Figure 3). These results suggest that (1) metabolic functions were diverse among the CAFs, (2) non-mitochondrial respiration and non-glycolytic acidification were decreased in CAFs, and (3) glycolysis relative to mitochondrial respiration was markedly enhanced in some CAFs.

**Figure 4:**
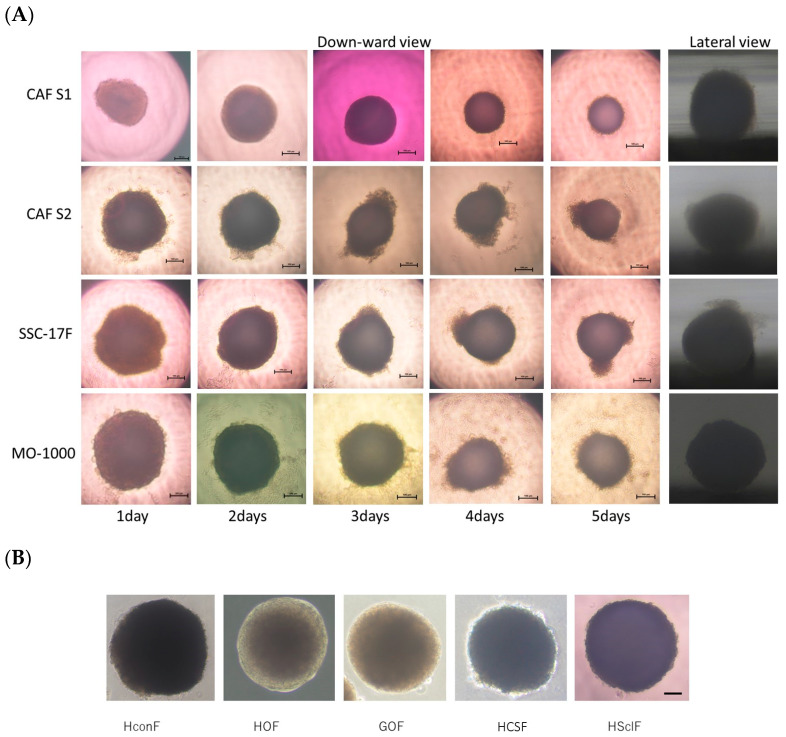
Representative PC (phase-contrast microscopy) images of 3D spheroids derived from 4 different CAFs and 5 different non-CAFs. Representative downward PC images at days 1 through 5 and lateral PC images at day 5 of 3D spheroids obtained from 4 different CAFs (CAFS1, CAFS2, SCC17F and MO-1000) are shown in panel (**A**). Alternatively, representative downward PC images of the fully matured non-CAFs (HconF, HOF, GOF, HCSF and HSclF) are shown in panel (**B**). Scale bar, 100 μm.

Since it is well known that CAFs are involved in the spatial microenvironments of cancers, a suitable experimental model that replicates such a spatial microenvironment would be highly desirable. For the purpose of this study, 3D spheroid cultures were employed using four different CAFs and five different non-CAFs. As shown in Figure 4, the four different CAFs all successfully formed 3D spheroids during the 5-day culture period, and their horizontal and lateral PC images indicated that the configuration of CAFS1 and MO-1000 was globe-shaped as was observed for the non-CAFs, but the shapes of CAFS2 and SCC F17 were slightly deformed.

**Figure 5:**
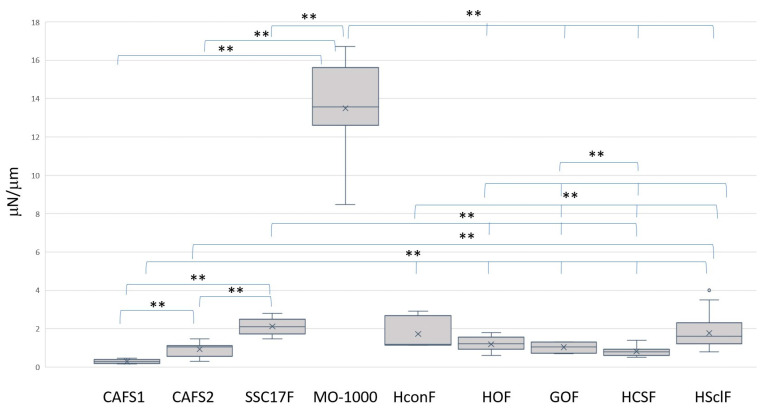
The mechanical stiffness of 3D spheroids obtained from 4 different CAFs and 5 different non-CAFs. Three-dimensional spheroids were prepared using 4 different CAFs (CAFS1, CAFS2, SCC17F and MO-1000) and 5 different non-CAFs (HconF, HOF, GOF, HCSF and HSclF) as above. Their mechanical stiffness was measured by a micro-squeezer, and the force required (μN) to compress a single spheroid to 50% of its original diameter (μm) during a micro-squeezer, and the force required (μN) to compress a single spheroid to 50% of its original diameter (μm) during a period of 20 s is plotted in the panel. ** *p* < 0.01.

To elucidate this further, the stiffness of the 3D spheroids was also compared among these four different CAFS and five different non-CAFs (Figure 5). Interestingly, substantial differences were also detected, and these differences were exclusively dependent on the degree of neighboring tumor differentiation as observed in the Seahorse cellular metabolic functions described above. That is, the 3D spheroid stiffness index levels, the force required (μN) to compress the spheroids to 50% of their original diameter (μm), of the non-CAFs were all within a range from 1 to 2. However, in contrast, the index value of MO-1000 was substantially higher and the values for CAFS1 and CAFS2 were significantly or relatively lower as compared with non-CAFs.

**Figure 6:**
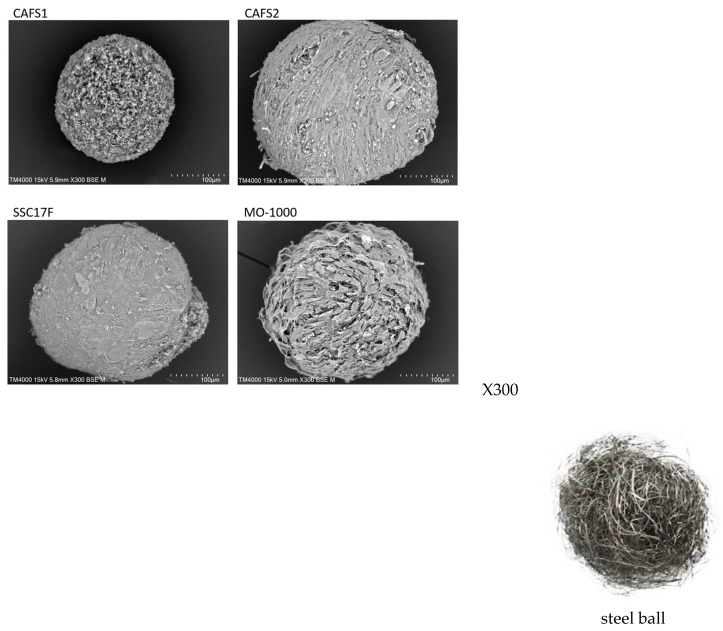
Representative SEM images of 3D spheroids derived from 4 different CAFs. Representative SEM images of the 3D spheroids obtained from 4 different CAFs (CAFS1, CAFS2, SCC17F and MO-1000) are shown. Scale bar, 100 μm. In addition, a representative image of the surface of the steel ball is shown.

To support such diversity in the stiffness of the 3D spheroids among the four different CAFs, their ultrastructure and intercellular binding properties were examined by scanning electron microscopy (SEM) and trypsin-induced dispersion, respectively. SEM analyses (Figure 6) demonstrated that the ECM deposits were quite sparsely distributed in the 3D CAFS1 spheroids, but such sparseness was diminished in the order of the 3D CAF2 spheroid and the 3D SCC17 F spheroid. Alternatively, the values for the 3D MO-1000 spheroids were markedly increased and developed, and resembled steel balls.

**Figure 7:**
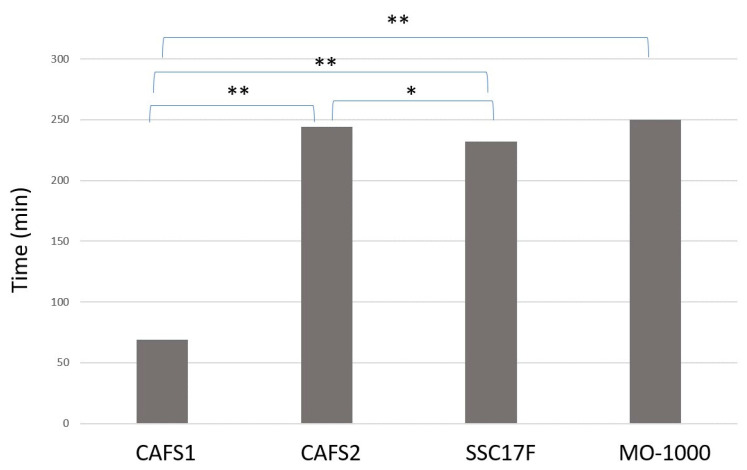
Trypsin digestion of 3D spheroids obtained from 4 different CAFs. Three-dimensional spheroids prepared using 4 different CAFs (CAFS1, CAFS2, SCC17F and MO-1000) were each treated with 0.25% trypsin until they were completely dispersed. Representative phase contrast microscopy images of 3D spheroids are shown (scale bar, 100 μm). Experiments were repeated in triplicate using fresh preparations (3D; *n =* 10 spheroids each). * *p* < 0.05, ** *p* < 0.01.

Alternatively, in the trypsin-induced dispersion experiments, the time required to completely disperse the cells of 3D spheroids in the presence of 0.25% trypsin was much faster for the CAFS1 (70 min) as compared with the other three CAFs (230–250 min) (Figure 7). Therefore, these collective results suggest that the biological characteristics of the CAFs and non-CAFs were significantly different, as well as among each of the CAFs.

**Figure 8:**
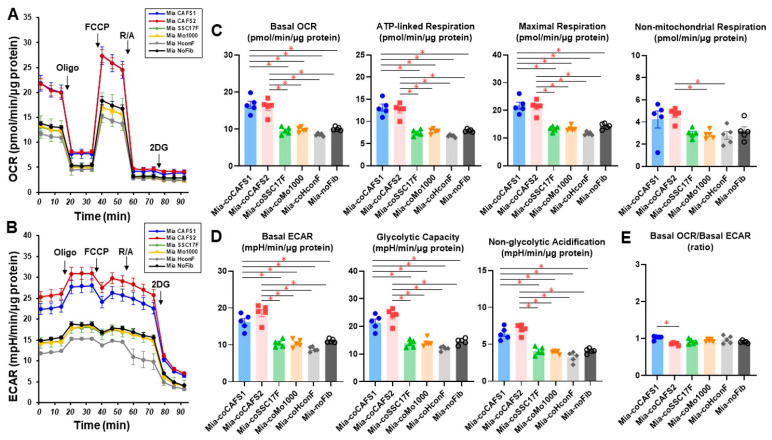
Measurement of the mitochondrial and glycolytic functions of pancreas ductal carcinoma cells MIA PaCa-2 co-cultured with four different CAFs or HconF, a non-CAF. A Seahorse real-time metabolic function analysis of 2D-cultured MIA PaCa-2 cells after co-culture with CAFS1, CAFS2, SCC17F, MO-1000 or HconF for 24 h. MIA PaCa-2 cells that were not co-cultured with fibroblasts are represented as MIA-NoFib. OCR (panel (**A**)) and ECAR (panel (**B**)) were measured at baseline and those with injections of sequential supplementation with a complex V inhibitor, oligomycin (Oligo), a protonphore, carbonyl cyanide-p-trifluoromethoxyphenylhydrazone (FCCP), complex I/III inhibitors, rotenone/antimycin A (R/A) and a hexokinase inhibitor, 2-deoxyglucose (2DG). Key parameters of mitochondrial functions and glycolytic functions are shown in panels (**C**,**D**), respectively. Basal OCR/Basal ECAR ratio is shown in panel (**E**). All experiments were performed using fresh preparations (*n =* 5). Data are expressed as mean or individual values and all error bars are displayed as SEM. * *p* < 0.05.

To investigate the potential CAF-inducible effects toward malignant tumors further, these four different CAFs or HconF (as a model of a non-CAF) were co-cultured with MIA PaCa-2 using a Transwell cell culture insert for 24 hrs, and the resulting samples were each subjected to a Seahorse cellular metabolic analysis (Figure 8). Various indices related to the OCR (panel C) and ECAR (panel D) for MIA PaCa-2 were significantly increased in the cases of CAFS1 or CAFS2, but this was not observed for the other CAFs or HconF, suggesting that the mitochondrial and glycolytic functions of MIA PaCa-2 were greatly influenced and altered by some populations of CAFs despite the fact that no effects caused by the non-CAF, HconF, were detected. However, as shown in panel E, the lower ratios of the basal OCR/basal ECAR of MIA PaCa-2 were due to the so-called “Warburg effects” [44], but were not affected by CAFs and HconF. Therefore, these results indicate that some of the CAFs were able to modulate the cellular metabolic functions of the co-cultured malignant tumors more efficiently as compared with non-CAFs. In addition, since the degree of neighboring tumor clinical stages as well as the TNM classification (Table 1, CAFS1 and CAFS2, T2N0M0, stage II; SCC17F and MO-1000, T4aN2bM0, stage IVA) were different, such effects induced by CAFs may be influenced by the degree of the clinical stage and the TNM classification of the originated neighboring tumors.

## 4. Discussion

The TSM has been recognized as one of the most important factors affecting the initiation, progression and invasion of a tumor, in which, as possible underlying mechanisms, CAFs, the major component of the TSM, are the responsible players due to them secreting various factors and ligands in addition to ECM proteins [12]. Since CAFs have extremely heterogeneous biological characteristics, it was not possible to distinguish between CAFs within the TSE and normal fibroblast CAFs in terms of their functional properties; CAFs are, in turn, defined by the expression of certain specific biological markers [16]. For example, various markers, such as αSMA, PDGFRα and FAP, which are expressed at high levels by CAFs, are widely used to isolate CAF populations. However, the specificity of most of these markers is quite low, and therefore, the issue of whether or not these markers can be used to universally verify CAFs, or merely a specific population within all CAF populations, continues to be questionable [16]. Therefore, to define a CAF population and their subpopulation, additional and rationale indices for use in evaluating CAFs themselves or inducible biological functions are needed. In the present study, the cellular metabolic states, and the efficiency of forming 3D spheroids, were studied in CAFs themselves along with their inducible biological characteristics, and the following observations were obtained: (1) The mRNA expression of positive and negative markers for CAFs or fibroblasts were established for our four fibroblasts, CAFS1, CAFS2, SCC17F and MO-1000 and they were confirmed to be CAFs; (2) although the mitochondrial and glycolytic functions were nearly identical between HOF and GOF, those of CAFs were significantly different as compared with a representative non-CAF, HconF; and (3) differences were found between CAFs and non-CAFs, and a similar diversity was found in the levels of CAFS1 and CAFS2, or SCC17F and MO-1000, as well as in terms of their physical aspects, i.e., the appearance and stiffness of their 3D spheroids and the co-culture-induced effects on the cellular metabolic functions of MIA PaCa-2. Based upon these results, we conclude that the cellular metabolic functions and physical characteristics of the 3D spheroids could be useful indices for identifying CAFs from fibroblasts as well as to differentiate CAFs into their subpopulations.

In contrast, regarding conventional in vitro 2D planar cell culture models, interest has arisen concerning the in vitro 3D cell culture models as being more physiologically relevant models for replicating the spatial local environments of malignant tumors. Therefore, such in vitro 3D cell culture models are now being more frequently used for testing the efficacy as well as suitable dosages of anticancer drugs [23,24,25,26,27]. Among the various in vitro 3D cell culture models [28,29], we focused to the simplest 3D drop culture method, and we successfully established various in vitro 3D spheroid models using non-cancerous cells [30,31] as well as cancerous cells [35,36]. During these investigations, since the subjected cells spontaneously formed droplets in the culture medium to form 3D spheroids, we speculated that the formation of such 3D spheroids may be caused by potential unknown biological factors. In fact, we found that almost all of the non-cancerous cells that we tested formed globe-shaped 3D spheroids [30,31], while in contrast, non-globe-shaped 3D spheroids were produced from most of the cancerous cells that we examined [35,36,38]. Furthermore, such cancer-cell-related 3D spheroids varied significantly among various cell lines with different clinical and pathological backgrounds even from the same origin [38,45]. Therefore, these findings also prompted us to speculate that 3D spheroid cultures may allow biological characteristics to be observed that were not observed in conventional 2D planar cell cultures. In fact, we found that adipogeneses of 3D 3T3-L1 spheroids were significantly enhanced as compared with those of 2D-cultured cells even though both were cultured under the exact same conditions except that different culture plates were used [37]. Therefore, based upon these collective findings, it was not surprising that the physical properties of the 3D CAF spheroids were quite different from those of non-CAFs as well as among CAFs.

In the cellular metabolic measurements conducted in this study, we found that the levels of metabolic parameters were markedly diverse among the types of CAFs, and that the ratios of basal OCR/basal ECAR of the four CAFs were also decreased as compared with a representative non-CAF, HconF. Because of the rapid glycolysis, even under aerobic conditions, the increase in glucose uptake and lactate secretion is recognized in cancerous cells, the so called the “Warburg effect” [46], and such a decrease in the basal OCR/basal ECAR ratios found in CAFs may be influenced by the neighboring tumor cells, a process that is referred to as the “reverse Warburg Effect” [47,48]. It is possible that the reverse Warburg effect can explain why tumor tissues can acquire fuel as an energy source via the transfer of energy from the host cells, CAFs, in the TSE. For example, cancerous cells can obtain lactate and pyruvate from the caveolin-1-deficient stromal fibroblasts for use as energy fuels in the mitochondrial tricarboxylic acid (TCA) cycle to enhance tumor proliferation [49]. Concerning the present co-culture experiments, the cellular metabolic functions of MIA PaCa-2 cells were markedly but differently modulated by CAFs as compared to HconF cells. That is, CAFS1 and CAFS2, in which the originated neighboring tumor was in the clinically early stage, induced substantial and similar increases in both mitochondrial and glycolytic functions, although these functions were not significantly altered in the SSC17F, MO-1000 and HconF cells. Such a metabolic change in Mia PaCa-2 cells that were co-cultured with CAFS1 and CAFS2 may also reflect a phenomenon that is referred to as the “reverse Warburg effect”, but it is noteworthy that the metabolic function of Mia PaCa-2 cells in the present study was measured in freshly-prepared medium with these CAFs being removed at the time of measurement. In other words, the metabolic changes induced by co-cultured CAFs cannot be explained by changes in metabolites alone, suggesting that cancer metabolism itself may have been “rewired”. As of the time of writing this paper, although the possible underlying mechanisms responsible for causing such CAF co-culture-induced alterations in the cellular metabolic functions in MIA PaCa-2 cells have not yet been elucidated, this represents possible proof of the existence of crosstalk between host tumor cells and the TSE. Indeed, non-mitochondrial respiration and non-glycolytic acidification, which typically reflect the activation of other cytoplasmic oxidases, pro-oxidant production and cellular proton production, were reduced in all CAFs compared to HconF cells, and the reduction in these indices may have been due to the presence of cancer cells. Alternatively, metabolites called “oncometabolites”, which are produced by cancer cells, may have contributed to the rewiring of the metabolic pathway [50]. Nevertheless, this observation provides support for the accumulating evidence that CAFs contribute to the acceleration in tumor growth and induce chemoresistance in pancreatic cancer [51]. As a result, targeting CAFs to improve pancreatic cancer therapies has hampered [52] the development of alternative strategies for suppressing CAFs. In this study, we provide new information on the metabolic reprogramming of CAFs and cancer cells in the setting of a co-culture system using a metabolic analyzer. The complete characterization of metabolic crosstalk in CAFs and cancer cells may provide clues for the development of novel treatments in the future. Furthermore, in our previous studies, we found that cellular metabolic functions were different among four different OSCCs and these functions were substantially modulated by the level of their pathological malignancy [45]. That is, as the pathological malignancy increased, oxygen utilization for ATP production in mitochondria and glycolytic capacity correspondingly increased, suggesting that malignant tumor cells have a high energy demand in order to continuously proliferate in OSCCs. Taking these observations into account, we speculate that CAFs may have the ability to compensate for the cellular metabolic states of co-cultured malignant tumors with CAFs since the cellular metabolic states of the two different CAF groups with different backgrounds in their clinical malignant stages as well as their pathology and inducible effects toward MIA-PaCa2 were different. However, this hypothesis is very speculative at present because both CAFs and non-CAFs that originated from different patients should each have unique clinical features and personal backgrounds. Therefore, additional investigations using CAFs obtained from various types of cancers as well as different clinical and pathological stages will be needed using a larger sample size. In addition, as further limitations in this study, we acknowledge that we were unable to assess the metabolic phenotype of the CAFs in 3D culture since the size and morphology of 3D-cultured CAFs were markedly different depending on the types of CAFs, as shown in Figure 6, resulting in different responses to injected reagents such as oligomycin and FCCP, thus making a comparative evaluation of metabolic functions between groups in 3D-cultured CAFs questionable and difficult. Furthermore, we wish to state that establishing a co-culture system using 3D-cultured spheroids may allow for the evaluation of the association of CAFs with cancer cells under more physiological conditions.

## 5. Conclusions

In conclusion, the findings observed in this study indicate that the cellular metabolic functions assessed by an extracellular flux analyzer and the physical properties of 3D-cultured spheroids can be valuable indicators for estimating potential biological diversity among various CAFs in addition to their identification using possible CAF markers.

## Figures and Tables

**Table 1:** Details of the CAFs.

CAF	Sex	Age	Tumor Site	Histological Type	TNM	Stage	Pathological Grade	YK Classification of Invasion
CAFS1	F	73	Tongue	OSCC	T2N0M0	II	2 (moderately differentiated)	4C
CAFS2	M	81	Tongue	OSCC	T2N0M0	II	2 (moderately differentiated)	4C
SCC17F	F	48	Tongue	OSCC	T4aN2bM0	IV A	3 (poorly differentiated)	4D
MO-1000	M	81	Gingiva	OSCC	T4aN2bM0	IV A	2 (moderately differentiated)	3

**Table 2:** Details of the non-CAFs established in our laboratory.

Non-CAFs	Surgically Dissected Tissue	Patients (Numbers)	Age Distribution	Sex	Passaging	Ref
HOF	orbital fatty tissue	non-Graves Orbitopathy (*n =* 5)	61–81	M4, F1	less than 3	[31,42]
GOF	orbital fatty tissue	Graves Orbitopathy (*n =* 5)	48–75	M4, F1	less than 3	[34]
HSSF	cornea	ocular injury (*n =* 2)	71, 77	M1, F1	less than 3	[33]
HSclF	sclera	rhegmatogenous retinal detachment (*n =* 7)	32–75	M4, F3	less than 3	[32]

Human orbital fibroblast (HOF), Graves’-disease-related HOF (GOF), human scleral stromal fibroblast (HSSF), human scleral fibroblast (HSclF).

## Data Availability

The data can be shared upon request.

## Supplementary Materials

The following supporting information can be downloaded at: https://www.mdpi.com/article/10.3390/cells12172160/s1, Table S1. Primers for qPCR.
