# Supplementary material for: Physical Properties and Cellular Metabolic Characteristics of 3D Spheroids Are Possible Definitive Indices for the Biological Nature of Cancer-Associated Fibroblasts
Source: Cells. 2023 Aug 28;12(17):2160. doi: 10.3390/cells12172160 (PMC10486986; doi:10.3390/cells12172160)
Supplement: Supplementary file 1 [file cells-12-02160-s001.zip › cells-2525465-supplementary.pdf]

Supplementary File
Table S1

FAP	Taq-man	GAAGTTGAA-GACCAGAT-TACAGC	GACCAG-TTCCAGATGCAAGG	/56-FAM/CCTCCA-TAG/ZEN/CAC-CAGCCCCATATG/3IAB-kFQ/	NM_004460	20-22
α SMA	Taqman	CTGTTGTAGGTGGTTTCATGGA	AGAGTTACcGAGTTGCCTGATG	/56-FAM/AGACCCTGT/ZEN/TCCAGCCATCCTTC/3IABkFQ/	NM_001613	8-9
FDGF α	Taqman	ATTCCTCTGCCTCACATTGAC	TTGATGAAGGTGGAACTGCT	/56-FAM/AGCTGTGTC/ZEN/TGTTCCTCTTGCCC/3IABkFQ/	NM_006206	22-23
VIM	Taqman	CAAGACCTGCTCAATGTTAAGATG	GTGAATCCAGATTAGTTTCCCTCA	/56-FAM/CGCCTTCCA/ZEN/GCAGCTTCCTGTA/3IABkFQ/	NM_003380	7-9
EPCAM	Taqman	TGTTGCTTGGAATTGTTGTGC	CCTATGCATCTCACCCATCTC	/56-FAM/CAGCCTTCT/ZEN/CATACTTTGCCATTCTCTTCT/3IABkFQ/	NM_002354	7-9
